# Books and Magazines

**Published:** 1903-03

**Authors:** 


### Books and Magazines.

**All books intended for review in the Texas Medical Journal must be sent to Associate Editor Dr. W. B. Russ, San Antonio, Texas.**

We have received from the publishers a copy of a book entitled, "The Practical Treatment of Stammering and Stuttering." The book is published by Geo. Andrew Lewis, 35-41 Adelaide street, Detroit, Mich., price, retail, $3.50. It can be purchased direct from the publishers or from any of the large houses that deal in medical publications.

---

TRANSACTIONS OF THE SOUTHERN SURGICAL AND GYNECOLOGICAL ASSOCIATION, VOLUME XIII.—Thirteenth session held at Atlanta, Ga., November 13, 14 and 15, 1900. Published by the Association, 1901. W. D. Haggard, M. D., Nashville, Tenn., Secretary.

SAUNDERS' QUESTION COMPENDS.—ATLAS AND EPITOME OF TRAUMATIC FRACTURES AND DISLOCATIONS.—By Prof. Dr. H. Helferich, Professor of Surgery at the Royal University, Griefswald, Prussia. Edited, with additions, by Joseph C. Bloodgood, M. D., Associate in Surgery, Johns Hopkins University, Baltimore. From the fifth revised and enlarged German edition. With 216 colored illustrations on 64 lithographic plates, 190 text-cuts, and 353 pages of text. Philadelphia and London: W. R. Saunders & Co., 1902. Cloth, $3.00, net.

Too much cannot be said in praise of the lithographic plates and numerous illlustrations contained in this number of Saunders' Medical Hand Atlas. For completeness and accuracy of portrayal of the conditions represented, these constitute by far the best collection of the kind that has yet appeared.

The text of the work is an epitome written and arranged to accompany the illustrations and it is satifactory in every particular.

The volume is a worthy addition to Saunders' Series of Hand

Atlases, and will be found an interesting volume to both students and practitioners.

